# Distinct Transcriptome Expression of the Temporal Cortex of the Primate *Microcebus murinus* during Brain Aging versus Alzheimer's Disease-Like Pathology

**DOI:** 10.1371/journal.pone.0012770

**Published:** 2010-09-16

**Authors:** Ronza Abdel Rassoul, Sabine Alves, Véronique Pantesco, John De Vos, Bernard Michel, Martine Perret, Nadine Mestre-Francés, Jean-Michel Verdier, Gina Devau

**Affiliations:** 1 Université Montpellier 2, Montpellier, France; Inserm U710, Montpellier, France; EPHE, Paris, France; 2 CHU Montpellier, Institute for Research in Biotherapy, Hôpital Saint-Eloi, Montpellier, France; 3 Service de Neurologie, Hôpital Ste Marguerite, Marseille, France; 4 UMR 7179 CNRS/MNHN, Brunoy, France; Federal University of Rio de Janeiro, Brazil

## Abstract

Aging is the primary risk factor of neurodegenerative disorders such as Alzheimer's disease (AD). However, the molecular events occurring during brain aging are extremely complex and still largely unknown. For a better understanding of these age-associated modifications, animal models as close as possible to humans are needed. We thus analyzed the transcriptome of the temporal cortex of the primate *Microcebus murinus* using human oligonucleotide microarrays (Affymetrix). Gene expression profiles were assessed in the temporal cortex of 6 young adults, 10 healthy old animals and 2 old, “AD-like” animals that presented ß-amyloid plaques and cortical atrophy, which are pathognomonic signs of AD in humans. Gene expression data of the 14,911 genes that were detected in at least 3 samples were analyzed. By SAM (significance analysis of microarrays), we identified 47 genes that discriminated young from healthy old and “AD-like” animals. These findings were confirmed by principal component analysis (PCA). ANOVA of the expression data from the three groups identified 695 genes (including the 47 genes previously identified by SAM and PCA) with significant changes of expression in old and “AD-like” in comparison to young animals. About one third of these genes showed similar changes of expression in healthy aging and in “AD-like” animals, whereas more than two thirds showed opposite changes in these two groups in comparison to young animals. Hierarchical clustering analysis of the 695 markers indicated that each group had distinct expression profiles which characterized each group, especially the “AD-like” group. Functional categorization showed that most of the genes that were up-regulated in healthy old animals and down-regulated in “AD-like” animals belonged to metabolic pathways, particularly protein synthesis. These data suggest the existence of compensatory mechanisms during physiological brain aging that disappear in “AD-like” animals. These results open the way to new exploration of physiological and “AD-like” aging in primates.

## Introduction

In EU, life expectancy is about 83 years for women and 76 for men, and it is expected to further increase. This trend will have major economic and social impacts due to the prevalence of age-related diseases. “Normal” age-related cognitive changes have been traditionally ascribed to cell loss in the brain. However, experimental evidence is now accumulating suggesting that synaptic rearrangement, rather than cell loss, causes critical age-related brain changes [1]–[3]. Most elderly individuals experience no major, or very limited functional impairment thus highlighting the human brain ability to compensate for potential cognitive decline. In other words, although aging may be a cause of cognitive alteration and is the main risk factor for Alzheimer's disease (AD) [4], they are clearly two separated processes. AD is not accelerated aging and therefore it is important to be able to distinguish physiological from pathological aging. The discovery of genes, which are differentially expressed in normally aging and AD brains, could provide a tool to identify biomarkers [5] that can differentiate between physiological and pathological brain aging and which might help to identify new therapeutic targets.

Non-human primates constitute a valuable tool for studies on brain aging because of their lifespan and their closeness to humans. We propose to take advantage of the grey mouse lemur, *Microcebus murinus*, which is a pro-simian primate whose lifespan in captivity can reach 12–13 years. In grey mouse lemurs, the 50% survival point occurs at 60 months (i.e., at the age of 5 years) and allows defining the adult and elderly part of the population [6]. Animals older than 5 years of age show desynchronized biological rhythms, motor activity and sleep-wake cycles [6], [7]. Importantly, during aging, some lemurs develop, like humans, pathognomonic signs of AD such as presence of ß-amyloid plaques [8] with a prevalence of 5% (data unpublished), tau protein aggregation [9] and cerebral atrophy [10]. Animals with cerebral atrophy present the most severe neuropathology associated with a progressive and consistent pattern of neuronal-glial alterations [10]. Moreover, this animal model can be used to investigate cognitive deficits [11]–[14].

We thus analyzed the gene expression profiles in the temporal cortex of young adult, healthy elderly and old lemurs with ß-amyloid plaques (“AD-like” group) in order to identify genes involved in physiological and pathological aging. Since *Microcebus murinus* microarrays do not exist, we decided to use Affymetrix human genome chips since recent studies have illustrated the feasibility of detecting non-human primate brain transcripts using human genome chips [15]–[18]. We chose the temporal cortex because this region is connected to the hippocampus and to the frontal cortex, which are critical structures for learning and memory and are altered in AD. Analysis of the microarray data indicates that the temporal cortex has different gene expression profiles in young, old and “AD-like” animals and that several genes are differentially expressed in healthy aged and “AD-like” animals. Specifically, genes involved in metabolism, particularly in protein synthesis, were up-regulated during physiological brain aging, whereas in “AD-like” brains genes involved in protein synthesis and nuclear activity were often down-regulated.

## Results

### Histological characterization of young adult, elderly and “AD-like” brain in *Microcebus murinus*

**Figure 1:**
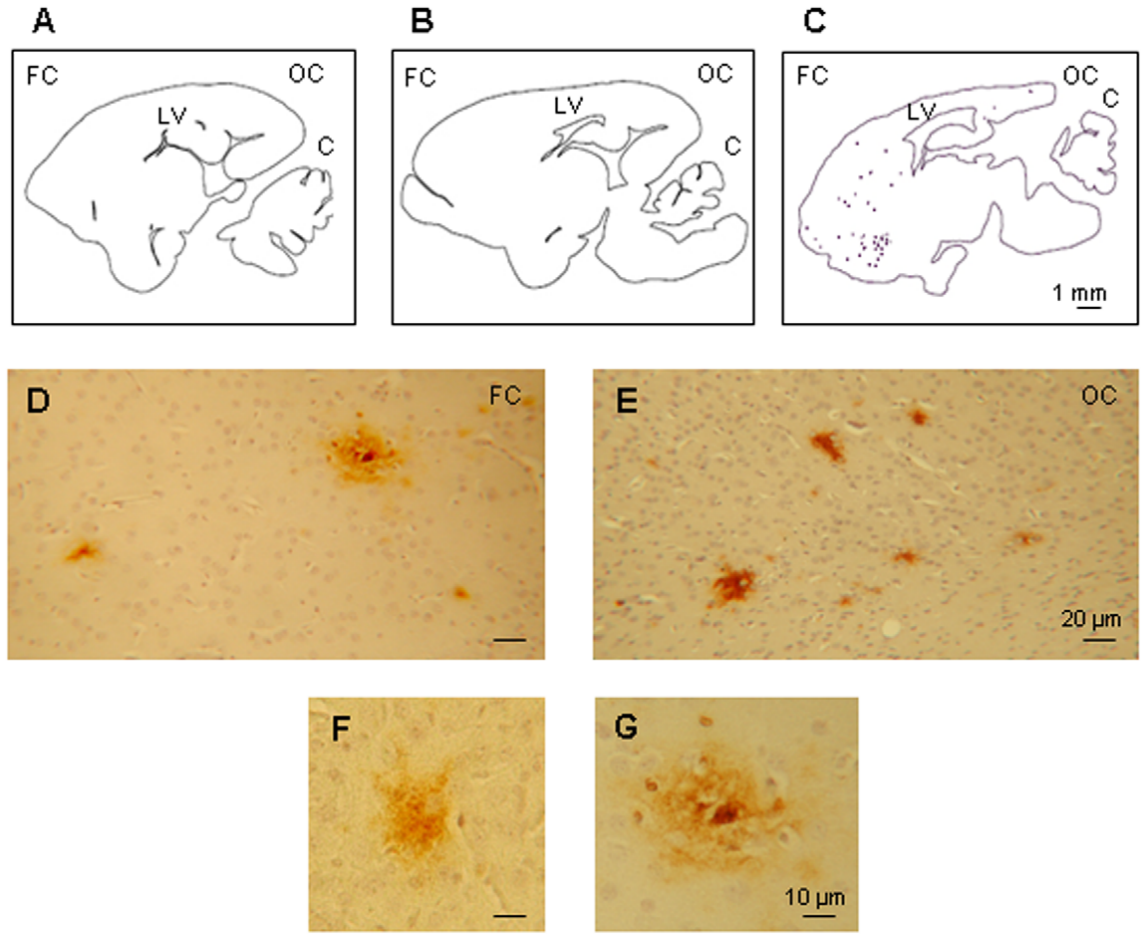
Brain sections and ß-amyloid plaques detection. Brain sagittal sections, localized at L 2.80 and L 3.00 from the median line according to the stereotaxic atlas of *Microcebus murinus* [40]. The contour of the brain slice was drawn with the Mercator software (ExploraNova, La Rochelle, France). ***A***. Young adult lemur (N^br^ 986) ***B***. Healthy old lemur (N^br^ 43) ***C***. AD-like old lemur (N^br^ 896). Frontal cortex (FC), occipital cortex (OC), cerebellum (C), lateral ventricle (LV). Amyloid deposits representative of the two “AD-like” lemurs, in the frontal cortex (D) and the occipital cortex (E). Higher magnification showing labeling of ß-amyloid plaques (F and G).

Before investigating the possible differences of gene expression in the temporal cortex of young adult, elderly and “AD-like” grey mouse lemurs, we compared the anatomical shape of the brains from the three groups (Fig. 1). We did not observe any difference in the thickness of the cerebral cortex and in the shape of the lateral ventricles between young (Fig. 1A) and healthy old animals (Fig. 1B). Conversely, in “AD-like” animals, we detected cortical atrophy, lateral ventricle dilatation (Fig. 1C) and ß-amyloid plaques (Fig. 1D,1E, 1F and 1G).

### Detection of transcripts with Affymetrix human genome chips

In order to assess the feasibility of using human genome arrays to study gene expression in the temporal cortex of *Microcebus murinus*, we first sequenced about 150 genes of the grey mouse lemur and compared them with their human homologues. They all presented a percentage of identity with their human counterparts that varied from 88% to 96% (data not shown). These results supported our choice of using the Affymetrix HG-U133 Plus 2.0 human microarray to investigate the pattern of gene expression in the temporal cortex of young adult, old and “AD-like” grey mouse lemurs (Table 1). The overall rate of genes with a “present” detection call (P) for each group was about 20%, with a slightly higher number of genes (9,456±1,378) detected in healthy aged animals, and a slightly lower number of genes (8,259±45) expressed in the “AD-like” group in comparison to the young adult group (8,914±1,482). Moreover, we noticed a lower SD in both “AD-like” animals, suggesting a better homogeneity of gene expression within this group. In human somatic tissues, which also included brain samples, the mean rate of genes with P call was 21278 (range: 7142–28389). Hence, although the percentage of genes detected by the HG-U133 Plus 2.0 microarray was lower in grey mouse lemur samples, it was still within the range of values observed in human samples.

**Table 1:** Genes detected in the temporal cortex of *Microcebus murinus*.

	Age (yr)	Males	Females	Number of detected genes
Young	1±0.1	2	4	8914.67±1482.68
Old	7.8±2.9	3	7	9456.20±1378.93
AD-like	10±3.3	0	2	8259.00±45.25
All		5	13	9142.67±1349.59

Transcripts detected in temporal cortex samples from young adult, old and “AD-like” animals by hybridization to the Affymetrix HG-U133 Plus 2.00 Array. The ages and the detected genes were expressed as mean ± SD.

### Analysis of age-dependent gene expression changes in the temporal cortex

**Figure 2:**
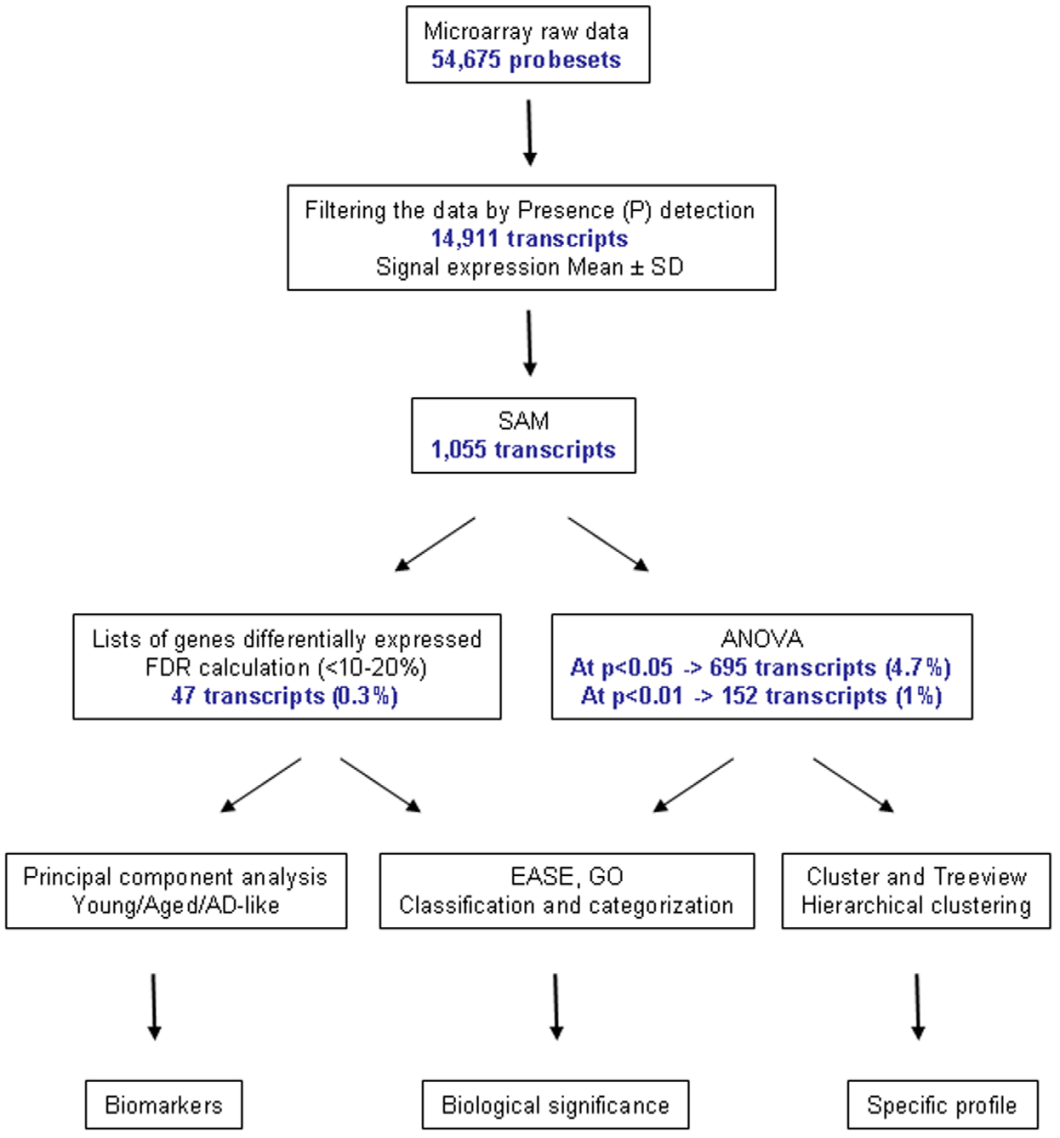
Schematic representation of the different analyses. Microarray data were filtered to detect the present transcripts (P). Then the filtered data were processed by SAM. ANOVA was performed with the (P) data and with the data sorted by SAM and the 47 P selected genes were analyzed by PCA. Finally, the 47 and the 695 genes were investigated by clustering and were classified by functional categories.

In order to characterize the expression profiles of adult, old and “AD-like” temporal cortex of *Microcebus murinus*, we first filtered the raw data (see Figure 2 for a schematic description for the analytical approach) and kept only the transcripts (n = 14,911) which had a “P” call in at least 3 samples. We then used the Significance Analysis of Microarrays (SAM) method to identify genes that were differentially expressed when comparing the three groups together or when assessing only healthy elderly and young or “AD-like” animals. The comparison of the three groups identified 1,055 transcripts with a false discovery rate (FDR) up to 45%. Since this risk of false positive was high, we assessed the significance of the changes in gene expression by ANOVA. The ANOVA analysis with a cut off at p<0.05 identified 695 genes with a significant gene expression change (i.e. 4.7% of expressed genes) and this number was reduced to 152 when the cut off was set at p<0.01 (i.e. 1% of the expressed genes) (Fig. 2 and Table S1).

On the other hand, comparison of the gene expression data from the young and the old group by SAM sorted 44 transcripts (FDR about 10%, Table 2), which were significantly up-regulated (fold change between 1.37 and 3.82) in healthy aging animals (Table 3). These genes are mainly involved in protein synthesis, mitochondrial metabolism, and nuclear regulation. Conversely, comparison of the gene expression data from “AD-like” and healthy old animals indicated that 40 genes were strongly down-regulated (fold change between −1.6 and −3.3) in the “AD-like” group (Tables 4 and 5), with the exception of *GK001* that was up-regulated. Among these genes, only a fraction plays a role in protein synthesis and nuclear regulation.

**Table 2:** Number of genes identified by SAM as differentially regulated in the temporal cortex of old animals.

Delta	Significant genes	FDR%
**0.76**	**6**	**0.00**
0.74	14	6.84
0.64	30	9.58
**0.64**	**44**	**10.88**
0.56	53	12.65
0.56	70	13.68
0.49	83	17.31
0.43	105	20.06
0.43	116	20.64
0.42	148	21.35
0.41	177	22.18
0.40	213	23.83
0.39	233	24.25
0.36	334	27.24
0.33	399	31.08
0.30	532	34.74
0.28	605	37.67
0.25	710	41.41

Delta table: number of genes with significant expression change in brain of aged lemurs detected by SAM analysis of expression data from temporal cortex samples of 10 healthy old lemurs and 6 young adults. The delta parameter is the threshold used to select genes that are significantly differentially expressed. The false discovery rate (FDR, expressed in %) is defined by the number of false positive genes divided by the number of genes identified as differentially expressed. Higher delta values lower the FDR but also reduce the number of selected genes. Delta table.

**Table 3:** Genes identified by SAM as differentially regulated in the temporal cortex of old animals.

Probesets	Name	FC	p-value	Function
205061_s_at	EXOSC9	1.77	****	Prot. Synthesis
0200936_at	RPL8	1.58	***	Prot. Synthesis
200817_x_at	RPS10	1.56	***	Prot. Synthesis
226131_s_at	RPS16	1.59	***	Prot. Synthesis
211487_x_at	RPS17	1.70	***	Prot. Synthesis
212270_x_at	RPL17	1.54	***	Prot. Synthesis
214003_x_at	RPS20	1.65	****	Prot. Synthesis
200834_s_at	RPS21	1.65	***	Prot. Synthesis
218830_at	RPL26L1	1.59	**	Prot. Synthesis
219762_s_at	RPL36	1.85	****	Prot. Synthesis
212863_x_at	CTBP1	1.47	**	Prot. Synthesis
200757_s_at	CALU	1.37	****	Prot. Maturation
222578_s_at	UBE1DC1	1.58	***	Proteolysis
201322_at	ATP5B	1.38	**	Mitoch. Metabolism
207507_s_at	ATP5G3	1.56	*	Mitoch. Metabolism
200818_at	ATP5O	1.49	**	Mitoch. Metabolism
201226_at	NDUFB8	1.63	**	Mitoch. Metabolism
**201227_s_at**	**NDUFB8**	1.65	***	Mitoch. Metabolism
35201_at	HNRPL	1.34	*	RNA related
218117_at	RBX1	1.59	***	Nuclear factor
226465_s_at	SON	1.72	***	Nuclear factor
204009_s_at	KRAS	1.38	***	Epigenetic
207721_x_at	HINT1	1.58	**	Epigenetic
1555961_a_at	HINT1	1.68	***	Epigenetic
224415_s_at	HINT2	1.47	***	Epigenetic
222540_s_at	HBXAP	1.38	***	Epigenetic
204745_x_at	MT1G	2.04	***	Cell cycle regulation
206461_x_at	MT1H	2.02	***	Cell cycle regulation
218206_x_at	SCAND1	1.45	*****	Cell proliferation
208864_s_at	TXN	1.56	***	Cell proliferation
**205352_at**	**SERPINI1**	1.50	***	Neurogenesis
232520_s_at	NSFL1C	1.48	***	Synapse
202670_at	MAP2K1	1.59	****	Kinase
226888_at	CSNK1G1	1.35	*****	Kinase
**217848_s_at**	**PP**	1.69	*****	Phosphatase
205809_s_at	WASL	1.80	**	Transduction
219392_x_at	FLJ11029	1.36	***	Unknown
**229302_at**	**MGC33926**	1.46	*****	Unknown
**230076_at**	**FLJ10156**	1.47	****	Unknown
225956_at	LOC153222	1.54	*	Unknown
218859_s_at	C20orf6	1.88	**	Unknown
241993_x_at	—	1.90	*****	Unknown
**218011_at**	**—**	1.57	*****	Unknown

List of 44 transcripts detected with a delta value set at 0.64 and a FDR of about 10%. Genes were classified according to their function: metabolism, nuclear activity, neurotransmission–synaptic plasticity, signaling pathways and unknown functions. P-value of the Student's t-test: 0.05 (*); 0.01 (**); 0.05 (***); 0.001 (****); 0.0005 (*****); FC: fold change. The 6 genes detected with 0% FDR were noted in bold.

**Table 4:** Number of genes identified by SAM as differentially regulated in “AD-like” temporal cortex samples.

Delta	Significant genes	FDR%
**0.46**	**2**	**0.00**
0.38	27	17.42
0. 36	37	19.06
**0.35**	**40**	**21.16**
0.28	52	23.51
0.24	84	30.79
0.24	94	29.51
0.23	110	30.78
0.21	172	35.27
0.20	208	35.72
0.20	244	36.61
0.19	341	37.78
0.19	460	40.07
0.17	569	42.15
0.17	765	43.89
0.16	836	45.28
0.15	968	48.92
0.14	1073	51.27

Delta table: number of genes with significant expression changes in aged animals detected by SAM analysis of expression data from temporal cortex samples from 2 “AD-like” lemurs and 10 healthy old animals.

**Table 5:** Genes identified by SAM as differentially regulated in “AD-like” temporal cortex samples.

Probesets	Name	FC	p-value	Function
211487_x_at	RPS17	−2.07	***	Prot. synthesis
200834_s_at	RPS21	−1.60	****	Prot. synthesis
225672_at	GOLGA2	−1.88	*	Prot. maturation
38710_at	OTUB1	−1.51	*	Proteolysis
**35201_at**	**HNRPL**	−1.61	******	RNA related
203752_s_at	JUND	−1.86	*	Transcription
227772_at	LATS1	−1.98	*	Transcription
200055_at	TAF10	−1.90	***	RNA related
209431_s_at	ZNF278	−2.53	******	Nuclear factor
218977_s_at	SECP43	−2.71	******	Nuclear factor
48580_at	CXXC1	−2.84	*	Nuclear factor
223132_s_at	TRIM8	−1.74	*	Nuclear factor
208676_s_at	PA2G4	−1.90	*	Cell cycle regulation
218034_at	TTC11	−1.65	**	Apoptosis
219888_at	SPAG4	−3.34	**	Cytoskeleton
203069_at	SV2A	−1.64	**	Synapses
225058_at	GPR108	−2.23	**	Synapses
209982_s_at	NRXN2	−2.59	***	Synapses
229309_at	ADRB1	−1.79	*	Synapses
206397_x_at	GDF1	−2.01	*	Growth factor
210185_at	CACNB1	−1.70	*	Ion channel
**222432_s_at**	**GK001**	2.65	******	Signal. Transd.
213108_at	CAMK2A	−1.65	******	Kinase
215903_s_at	MAST2	−2.22	*****	Kinase
228302_x_at	CaMKIINalpha	−3.09	***	Kinase
209945_s_at	GSK3B	−1.97	*	Kinase
200822_x_at	TPI1	−1.87	***	Phosphate metabol.
33197_3_at	GAPD	−1.33	*	Phosphate metabol.
225519_at	PPP4R2	−2.08	******	Phosphatase
227325_at	LOC255783	−5.11	******	Unknown
228935_at	LOC283400	−5.20	******	Unknown
210408_s_at	CPNE6	−2.06	******	Unknown
217033_x_at	—	−1.68	*****	Unknown
1554429_a_at	DMWD	−1.87	****	Unknown
219392_x_at	FLJ11029	−1.79	****	Unknown
48106_at	—	−2.01	***	Unknown
219910_at	HYPE	−1.57	*	Unknown
207435_s_at	SRRM2	−1.97	*	Unknown
218089_at	C20orf4	−1.92	*	Unknown
218429_s_at	FLJ11286	−1.72	*	Unknown

List of the 40 transcripts sorted using a delta set at 0.35 and a FDR of 21%. All were down-regulated in AD, but for *GK001*. Genes were classified according to their function: metabolism, nuclear activity, neurotransmission–synaptic plasticity, signaling pathways and unknown functions. FC: fold change; P value: 0.05 (*); 0.01 (**); 0.05 (***); 0.001 (****); 0.0005 (*****). The 2 genes detected with 0% FDR were noted in bold.

When for the comparison of the three groups a more stringent delta cutoff was chosen (from 0.058 to 0.03) the number of genes identified as differentially expressed in the three groups was reduced to 47 and among them were included the 28 genes that had been already sorted by the two SAM analyses using the data from young and healthy old animals or healthy old and both “AD-like” animals (Tables 6 and 7).

**Table 6:** SAM analysis of the expression data from the three groups.

	Probesets	Name	FCA/Y	p-value	FCAD/Y	p-value	FCAD/A	p- value	Functions
A	226131_s_at	RPS16	1.59	***	1.19	NS	−1.34	*	prot synthesis
	214003_x_at	RPS20	1.65	****	1.03	NS	−1.60	***	prot synthesis
	218830_at	RPL26L1	1.59	***	1.26	*	−1.26	*	prot synthesis
	219762_s_at	RPL36	1.85	****	−1.02	NS	−1.88	*	prot synthesis
	200757_s_at	CALU	1.37	*****	−1.07	*	−1.47	*****	prot maturation
	201227_s_at	NDUFB8	1.65	****	1.20	**	−1.37	****	mitoch metabol.
	218206_x_at	SCAND1	1.45	*****	−1.04	NS	−1.51	***	cell prolif.
	207721_x_at	HINT1	1.58	***	1.13	NS	−1.40	NS	Epigenetic
	224415_s_at	HINT2	1.47	***	−1.04	NS	−1.53	*****	Epigenetic
	222540_s_at	HBXAP	1.38	***	1.09	NS	−1.27	**	Epigenetic
	204009_s_at	KRAS	1.38	***	1.04	NS	−1.32	*	Epigenetic
	202670_at	MAP2K1	1.59	*****	1.21	NS	−1.32	NS	Kinase
	217848_s_at	PP	1.69	*****	1.34	***	−1.26	*	Phosphatase
	230076_at	FLJ10156	1.47	*****	1.10	NS	−1.34	***	Unknown
	241993_x_at	—	1.90	****	1.35	NS	−1.41	NS	Unknown
	218011_at	—	1.57	*****	1.09	NS	−1.45	NS	Unknown
B	223132_s_at	TRIM8	−1.06	NS	−1.85	*	−1.74	*	nuclear fact.
	222432_s_at	GK001	−1.19	NS	2.23	******	2.65	*****	signal transd.
	213108_at	CAMK2A	1.42	*	−1.16	****	−1.65	*****	Kinase
	209945_s_at	GSK3B	1.43	*	−1.38	NS	−1.97	*	Kinase
C	211487_x_at	RPS17	1.70	****	−1.22	NS	−2.07	***	prot synthesis
	200834_s_at	RPS21	1.65	****	−1.03	NS	−1.60	****	prot synthesis
	35201_at	HNRPL	1.34	*	−1.20	*	−1.61	*****	RNA related
	219392_x_at	FLJ11029	1.36	***	−1.32	NS	−1.79	****	Unknown

Genes with significant expression changes identified when data from the cortex samples of young (Y), healthy aged (A) and “AD-like” (AD) were compared. FC: Fold Change. These genes have been already sorted as regulated by age (see Table 3); B: genes already identified as regulated by AD (Table 5); C: genes regulated by age and by AD (Table 3 and 5).

**Table 7:** SAM analysis of the expression data from the three groups.

Probesets	Name	FCA/Y	p-value	FCAD/Y	p-value	FCAD/A	p- value	Functions
200029_at	RPL19	1.69	***	1.07	NS	−1.58	NS	prot synthesis
222991_s_at	UBQLN1	−1.27	NS	1.61	**	2.04	***	Proteolysis
204528_s_at	NAP1L1	1.09	NS	2.15	******	2.34	*****	RNA related
213470_s_at	HNRPH1	−1.52	***	−1.45	*	1.05	NS	RNA related
204847_at	ZBTB11	1.34	**	2.17	******	1.62	*****	nuclear fact.
203138_at	HAT1	1.17	NS	2.14	**	1.83	**	Epigenetic
203845_at	PCAF	1.13	NS	1.93	NS	1.71	NS	Epigenetic
202260_s_at	STXBP1	1.08	NS	1.50	******	1.40	*****	Synapse
214441_at	STX6	−1.67	*****	−1.50	**	1.12	NS	Synapse
232520_s_at	NSFL1C	1.48	****	1.20	**	−1.23	*	Synapse
226683_at	SNAG1	1.01	NS	1.51	******	1.50	*****	Synapse
205352_at	SERPINI1	1.50	***	1.64	*	1.09	NS	Neurogenes.
222960_at	CACNA1H	−2.04	******	−1.81	*	1.13	NS	Ion channel
223208_at	KCTD10	−1.86	***	1.52	******	2.83	*****	Ion channel
211302_s_at	PDE4B	−2.29	***	−4.68	***	−2.05	NS	Phosphatase
1563674_at	SPAP1	1.68	NS	4.78	***	2.84	**	Phosphatase
218021_at	DHRS4	−1.49	*****	1.10	NS	1.64	**	Dehydrogen
229302_at	MGC33926	1.46	******	1.28	***	−1.14	*	Unknown
65635_at	FLJ21865	−1.30	***	−1.43	***	−1.10	NS	Unknown
215672_s_at	KIAA0828	−1.72	******	−1.66	NS	1.04	NS	Unknown
48106_at	—	−1.18	NS	−2.37	****	−2.01	***	Unknown
240835_at	—	1.16	NS	2.26	******	1.95	*****	Unknown
1553705_a_at	—	−1.50	***	−1.07	NS	1.41	***	Unknown

Genes with significant expression changes identified when data from the cortex samples of young (Y), healthy aged (A) and “AD-like” (AD) were compared. FC: Fold Change. These genes have been identified by SAM using the data from the three groups.

**Figure 3:**
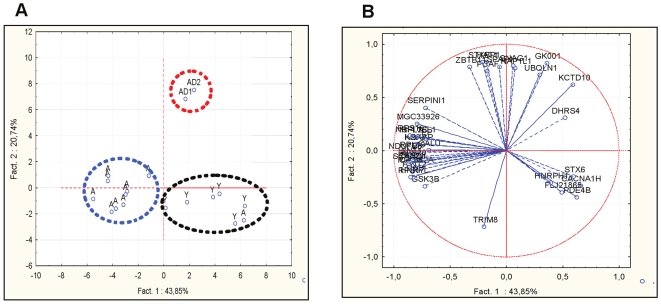
Principal component analysis of the 47 transcripts that are differentially regulated in aging or “AD-like” brain detected by SAM. **A**. Projection of the individuals shows that healthy aged animals can be discriminated from the young ones (axis 1), and “AD-like” can be differentiated from healthy old animals (axis 2). **B**. Projection of the genes. Genes located to the left of axis 1 characterize healthy aged animals, whereas genes located at the top of axis 2 characterize the “AD-like” group. Young (Y), aged (A) and “AD-like” (AD) animals.

Principal component analysis of the 47 genes, which were identified as differentially expressed in the three groups by SAM, confirmed that they could clearly separate young, healthy old and “AD-like” animals (Fig. 3). The first component represented the gene expression signature with a score of 64.59% of the variance in the three groups. Healthy aged animals could be differentiated from the young ones with a score of 43.85% (Fig. 3A, axis 1), and the “AD-like” group could be distinguished from the healthy old group with a score of 20.74% (Fig. 3A, axis 2). Genes with a high correlation coefficient were considered to be important for discriminating the three groups (Fig. 3B). Genes located to the left of axis 1 characterized the group of healthy aged animals. Most of them are involved in protein synthesis (*RPS16*, *17*, *19*, *20*, *21*, *RPL26L1* and *36*), mitochondrial metabolism (*NADH dehydrogenase complex*, *NDUFB8*) or in the regulation of transcription (*SCAND1*, *HNRPL*, *HINT1-2*, *KRAS*). Genes located at the top of axis 2 characterized the “AD-like” group. Some of these genes play a role in neurotransmission (*STXBP1*), intracellular trafficking (*SNAG1*), regulation of transcription (*ZBTB11*) or in epigenetic processes (*HAT1*). However, also genes, which are not yet reported as having brain-related functions, such as *SPAP1*, which is expressed in mature lymphocyte B cells, contributed to delineate the “AD-like” group. Calumenin (CALU), a protein which is involved in the regulation of vitamin K-dependent carboxylation of multiple amino-terminal glutamate residues and normally is present at very low levels in the brain, also was up-regulated in the 2 “AD-like” animals. Others encode proteins that are detected in neurofibrillary tangles, such as Ubiquilin-1 (UBQLN1), which promotes the accumulation of uncleaved PSEN1 and PSEN2 that are involved in AD pathology.

The 47 genes sorted by SAM were included in the 695 genes identified by ANOVA as differentially expressed in the three groups. Among these 695 genes, the expression of 350 was modified by age, the expression of 427 by the “AD-like” pathology (Table 8) and some by both. Among the 350 genes modified by age, 209 were up-regulated and 141 down-regulated, and among the 427 genes modified by the pathology, 250 were up-regulated and 177 down-regulated. Moreover, 28% of the genes that were differentially regulated in “AD-like” samples showed a similar change in expression also in healthy old animals, whereas 72% showed an opposite change of expression in comparison to the healthy old group. We also analyzed the expression data by taking into account only the females. In this case the number of transcripts with significant changes was higher: 1,079 instead of 695 because of less variance due to a better homogeneity of the populations.

**Table 8:** Genes that are differentially regulated during physiological or pathological aging sorted by ANOVA.

Comparison	Young *vs* Old	AD-like *vs* Old
Up-regulation	209	250
Down-regulation	141	177
All	**350**	**427**

**Figure 4:**
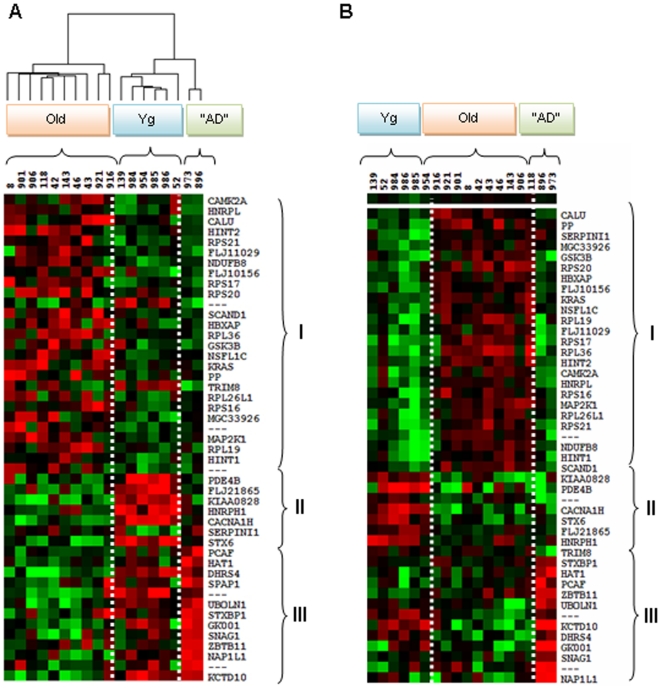
Transcriptional profiles in the temporal cortex of *Microcebus murinus*. Hierarchical clustering obtained with the 47 genes sorted by SAM. The transcriptional profiles of the temporal cortex of the 18 *Microcebus murinus* (i.e., 6 young adults (Yg), 10 old lemurs (Old) and 2 “AD-like” (AD)) showed three distinct profiles for the 3 groups. Each profile could be separated in three distinct regions which were conserved in the three groups. (A) Dendogram and clustering showing that the animals were clustered by age or by pathology. (B) Hierarchical clustering of lemurs according to age and pathology. The clusters show that the expression of some genes in “AD-like” lemurs is similar to that in young animals. The profiles of healthy elderly animals are drastically different from those of the two “AD-like” lemurs. In the three different parts: I- genes that are down-regulated in aging and in “AD-like” animals; II- genes that are up-regulated with young adults but are down-regulated in “AD-like” animals; III- genes that are down-regulated in old and up-regulated in “AD-like” animals. Red: over-expressed genes; green: down-regulated genes; black: genes without expression changes.

**Figure 5:**
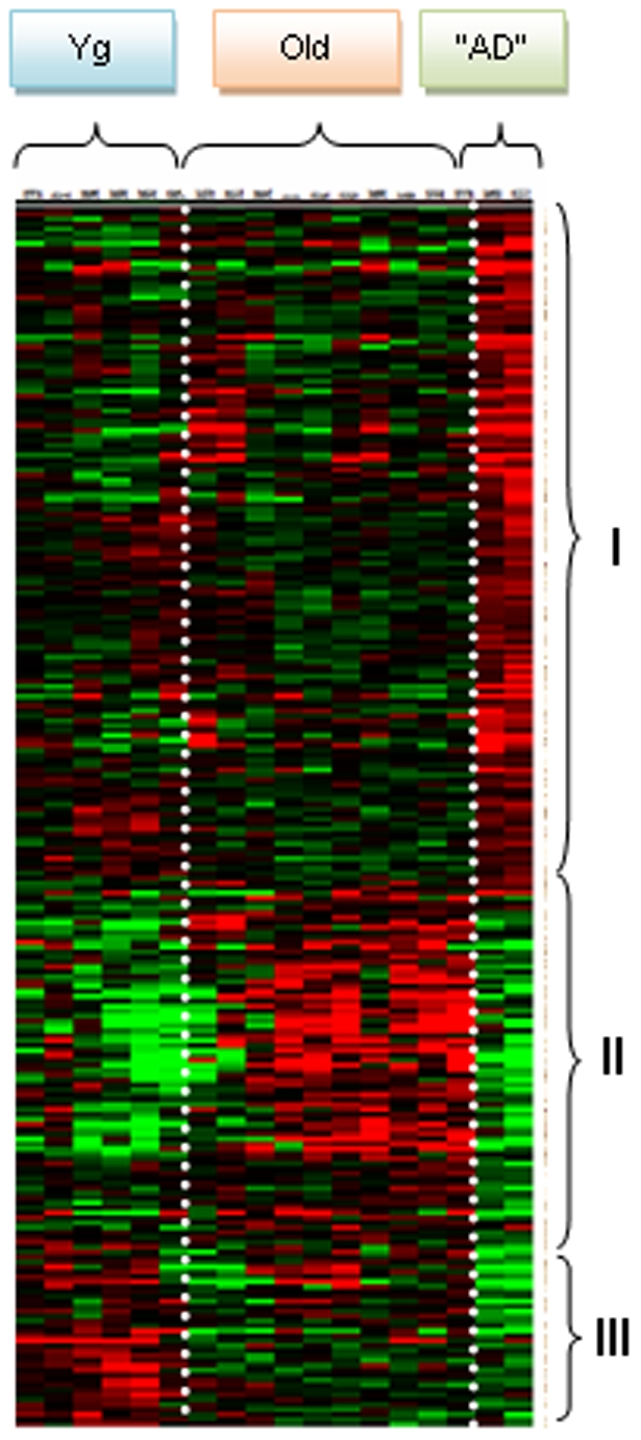
Transcriptional profiles in the temporal cortex of *Microcebus murinus*. Hierarchical clustering obtained with the 695 genes sorted by ANOVA. The transcriptional profiles of the 18 *Microcebus murinus* (i.e., 6 young adults (Yg), 10 old lemurs (Old) and 2 “AD-like” (AD)) showed 3 distinct regions: I- genes that are up-regulated in “AD-like” temporal cortex; II- genes that are down-regulated in “AD-like” temporal cortex and preferentially up-regulated in aged cortex; III- genes that are down-regulated in “AD-like” and preferentially up-regulated in young adult cortex. In red are shown genes that are over-expressed, in green genes that are under-expressed and in black genes without expression changes.

Finally, hierarchical clustering indicated that each group had a distinct profile whatever the statistical test (SAM (Fig. 4) or ANOVA (Fig. 5)) used to sort and select the genes and that each profile was homogenous within the specific group, particularly in the “AD-like” animals. Hierarchical clustering identified clusters of genes that were down-regulated in old and up-regulated in “AD-like” animals and genes, which were up-regulated in young adults and down-regulated in old and “AD-like” animals, in good agreement with the PCA data and also with the ANOVA analysis that showed opposite changes (Fig. 4II; 5II) and similar changes (Fig. 4 I; 5III) in gene expression.

### Functional categorization and sexually dimorphic changes in aged *Microcebus murinus* males and females

**Figure 6:**
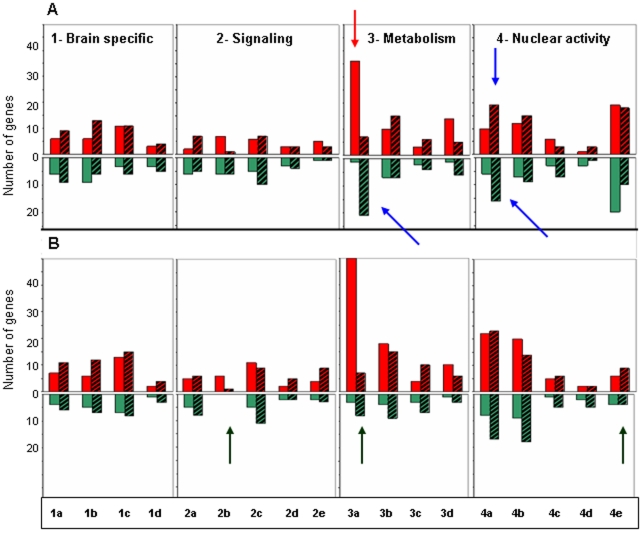
Gene changes in relation to their cellular function identified by gene ontology. The 695 genes sorted by ANOVA were classified into four main modules: 1- genes involved in brain plasticity [1a-neurotransmission, 1b-neurogenesis, 1c-adhesion and extracellular matrix, 1d-cytoskeleton]; 2- genes involved in transduction and signaling [2a-ion channels, 2b-kinases, 2c-phosphatases, 2d-transferases and 2e-growth factors]; 3- genes involved in metabolism and catabolism [3a-protein synthesis and maturation, 3b-proteolysis, 3c-glucidic and lipid metabolism and 3d-mitochondrial metabolism]; 4- genes involved in nuclear activity [4a-nuclear factors, 4b-transcription regulation, 4c-cell cycle regulation, 4d-apoptosis and 4e- epigenetic control]. Red bars represent genes that are up-regulated and the green bars genes that are down-regulated in the temporal cortex of aging animals; hatched red bars represent genes that are up-regulated and hatched green bars genes that are down-regulated in the temporal cortex of “AD-like” lemurs. ***A***. Classification with males and females together. ***B***. Classification with females only. In aging animals genes involved in protein synthesis were more frequently up-regulated (red arrow), and the most important differences between aging and “AD-like” profiles concerned genes that have a role in protein synthesis and nuclear activity (blue arrows).The major differences between A and B are indicated by black arrows.

By assessing the relative changes in expression within gene ontology categories, we could classify the differentially expressed genes according to their cellular functions and distribute them in 4 main modules: 1- Brain plasticity; 2- Signaling pathways; 3- Metabolism and protein synthesis; 4- Nuclear activity (Fig. 6 and Table S2_gene functional categorization). Most of the genes that were up-regulated with age and down-regulated in “AD-like” animals (especially those involved in protein synthesis) belonged to the “metabolism and protein synthesis” module (Fig. 6A, module 3a). Nuclear factors (Fig. 6A, module 4a) were differentially expressed particularly in the “AD-like” group. Analysis of the data from the 2 females only showed that the general profiles were maintained when compared with the data from all animals (Fig. 6B). Nevertheless, the number of genes that were up-regulated with age was higher, whereas the number of down-regulated genes involved in signaling transduction (Fig. 6B, module 2b), metabolism (Fig. 6B, module 3a) and in epigenetic regulations (Fig. 6B, module 4e) was lower.

## Discussion

*Microcebus murinus* is a useful primate model of cerebral aging and AD. We already showed that, not only they display pathognomonic lesions for AD and as observed in humans, both lemurs with AD-like pathology presented cortical atrophy. Recent studies have shown that brain measurement by MRI could be an important biomarker of degeneration like in the case of AD [19], [20]. They also represent a tool for anti-amyloid vaccination studies [21], MRI studies [10] and for developing cognitive tests [12], [14]. Therefore, to further develop the potentialities of this model, we wanted to identify in the temporal cortex of *Microcebus murinus* modifications in gene expression that were associated with physiological or pathological aging. Using human genome microarrays the overall rate of gene detection was about 20%. This value could appear to be rather low, but it has to be compared with other heterologous (*Rhesu*s monkey with human microarrays) or homologous (human with human microarray) studies where only 30–35% and 30–40% of genes were detected ([18]; JDV, personal communication). Our results also confirm earlier works [15]–[18] that human chips can be used in non-human primates.

Among more than 14,000 genes, we have identified 695 genes (around 4.9%) with significant expression changes in young, aged and “AD-like” animals. Analysis of the cortex transcriptome from human brain samples at different ages indicates that the expression of about 400 genes among 11,000 (around 3.6%) is significantly changed [22]. Our results are in the same order of magnitude.

We then compared the list of genes that changed in the human frontal cortex sorted by Lu et coll. (2004) with the genes changing in the lemurian temporal cortex. We found 13 common genes (out of 150) and also 58 (out of 150) that belong to the same family and have similar biological function (e.g. VAMP1/VAMP2 or Synapsin II and synapsin IIb). Most of the genes that are differentially expressed in both works appeared to be involved in the synaptic transmission (8/15), Ca^2+^ homeostasis (5/10), cAMP signalling (2/2)) and in transcription (6/8). In the study by Lu and coll. the expression of these genes was modified by age, whereas in our study, they are modified by age and/or by pathology.

The same applies to the fold changes that varied between 1.5 and 3. Moreover, analysis of the microarray data indicates that each group of animals (young adult, healthy old, and “AD-like”) has a distinctive gene expression profile in the temporal cortex, a structure involved in integrative functions such as memory. Previous studies have already concluded that the expression of many genes is modified during aging (reviewed in [2], [23], [24]. Specific profiles in relation with age were observed in human brain [22], [25] and in *Rhesus* monkey brain, particularly in the corpus callosum [18] and in the frontal cortex [25]. Interestingly, each brain region of adult or healthy elderly subjects had a distinct gene expression profile [26]–[28]. Altered gene expression was also observed between healthy elderly individuals and AD patients, for review [29], particularly in the hippocampus [30], [31] and in neurons of several brain structures [27].

Our results support the hypothesis that the events occurring during physiological brain aging and AD development are distinct. However, an important issue is to understand the transition from physiological to pathological brain aging which leads to neurodegenerative disorders. We observed that many of the genes up-regulated in healthy aged animals are involved in protein synthesis such as the genes coding for ribosomal proteins. These results support the idea that physiological aging, at least in the brain, is associated with permanent compensatory effects via increased protein synthesis. Conversely, these genes were down-regulated in the 2 “AD-like” animals, indicating a failure to compensate a decline in the metabolic activity in the brain of animals with amyloid deposits. Moreover, the expression of *UBQLN1*, an ubiquitin-like protein that plays a role in the regulation of the degradation of proteins by proteasome [32], was significantly decreased in both “AD-like” animals. This observation is important as accumulation of misfolded proteins, which are not degraded by the proteasome, may be involved in the development of neurodegenerative diseases. The expression of other genes that are related to the regulation of neural plasticity, an important process involved in brain aging [3], [33], also was changed in “AD-like” animals in comparison to healthy old ones. Particularly, *GRIN1*, the NR1 subunit of the NMDA glutamate receptor, and *GRIA1*, a subunit of the AMPA glutamate receptor, which activate pathways involved in the induction of long-term potentiation (LTP) were markedly down-regulated. AD-related pathologic processes lead to synaptic loss and dysfunction, particularly of glutamatergic transmission [34], [35] that can cause inhibition of LTP [36] as it has been shown also in rodents following acute treatment with Aβ oligomers [37]. Moreover, recent data have also demonstrated that changes in the expression of nuclear proteins, which induce epigenetic modifications of chromatin, play an important role in regulating synaptic plasticity and memory processes [38]. In our study, we observed that several genes implicated in epigenetic processes and transcription, such as histone acetyltransferases (*HAT1*, *PCAF*), histidine triad nucleotide binding proteins (*HINT1*, *HINT2*) and the cAMP responsive element binding protein (*CREB1*), were down-regulated during physiological and pathological brain aging. Conversely, *KRAS* and *HBXAP*, which mediates nucleosome assembly and chromatin remodeling, were up-regulated in healthy aged *Microcebus murinus*. Finally, the expression of *CLU* (*APOJ*, *Clusterin*), which is considered as a risk factor for AD [39], [40] was significantly increased in both “AD-like” animals.

Our data also indicate that changes in gene profiles during aging in *Microcebus murinus* temporal cortex might show gender-specificity, suggesting that brain continues to present sexual differences during the entire life. This observation needs, however, to be confirmed since both “AD-like” animals were females. Nevertheless, sexual dimorphism in gene expression in aging human brain has been reported by Berchtold et al. [28] and in primate brain by Reinius et al. [41], whereas Lu and colleagues [22] did not evidence significant gender differences in the transcriptomic profiles of the human frontal cortex.

Overall, our expression data support the relevance of *Microcebus murinus* as a valuable non-human primate model for studying molecular brain changes in relation with age and neurodegenerative diseases such as AD.

## Materials and Methods

### Animals

The *Microcebus murinus* animals were all born in captivity in our breeding colony (CECEMA, University of Montpellier 2). They were divided into three groups: the first (young animals) included six 1 year/old adults (4 females and 2 males), the second (healthy old animals) included ten 8 year/old animals (7 females and 3 males), and the third (“AD-like” animals) two old females with cerebral ß-amyloid plaques. About 5% of old lemurs in our colony develop ß-amyloid plaques in the brain (unpublished data). Animals were euthanized with 150 mg/Kg ketamine. Animal care was in accordance with institutional guidelines and the animal protocol was approved by the Ethic Committee, Comité Régional d'Ethique pour l'Expérimentation Animale - Languedoc Roussillon (authorization #CE-LR-0816, 2008) in accordance with the European Community Council directives of November 24, 1986.

### Brain samples preparation

After euthanasia, each brain was cut in half along the midline. The right hemisphere was used for the microarray experiments and the left one for immunohistological experiments. For transcriptional analysis, the right hemisphere was dissected and cortex samples, weighing 25–35 mg, were flash-frozen and stored at −80°C until use. The left hemisphere was fixed in paraformaldehyde and embedded in paraffin as previously described [8], [14] and 6 µm serial sagittal sections were prepared.

### Immunohistochemistry

Presence of amyloid-ß-42 (Aß42) peptide aggregates was assessed using the rabbit polyclonal FCA3542 antibody (Calbiochem, Merck-bio, Germany). Sections were deparaffinized and hydrated through ethanol gradient. After formic acid pretreatment, sections were pre-incubated with 10% goat serum in Tris-buffered saline for 30 min. Then, sections were incubated with anti-Aß42 antibodies (1∶1000) at 4°C overnight. Immunological complexes were detected with biotinylated immunoglobulins (1∶1000) and horseradish peroxidase-labelled avidin (Vector Laboratories, Burlingame, VT). Immunoreactivity was revealed with 0.005% diaminobenzidine tetrahydrochloride (DAB, 0.35 mg/mL, Sigma, St Louis, MO). The primary antibody was omitted in the negative control and sections from *Microcebus murinus* brain specimens known to contain ß-amyloid plaques were used as positive controls. The *Microcebus murinus* brain atlas was used to localize the brain area of each section [42].

### RNA isolation and Affymetrix GeneChip processing

Total RNA was extracted from each temporal cortex sample (25–35 mg) using the RNeasy mini kit (Qiagen, Santa Clarina, CA) according to the manufacturer's protocol. The quality of each purified RNA sample was checked with a NanoDrop Agilent BioAnalyzer (Agilent Technologies, Santa Clara, CA). An average of 2 µg of total RNA from each cortex sample was prepared for hybridization with Affymetrix HG-U133 Plus 2.00 GeneChip (Santa Clara, CA) according to the manufacturer's protocol. This microarray contains 54,656 probes corresponding to 47,400 human transcripts. At the end of the experiment, the hybridized probes were scanned at a resolution of 3 µm in a confocal scanner (Affymetrix GeneChip Scanner 3000 7G). Affymetrix microarrays were processed in the Microarray Core Facility of the Institute of Research of Biotherapy, CHRU-INSERM-UM1 Montpellier (http://irb.chu-montpellier.fr/).

### Affymetrix GeneChip data analysis and filtering

Data were normalized with the GeneChip Operating Software (GCOS) algorithms. Then, the converted digital intensity values were stored as image data files and converted into cell intensity files using the Microarray Affymetrix Software 5.0 (MAS 5). MAS5 labels each transcript as “present” (P), “marginal” (M) or “absent” (A) by taking into account the average difference value calculated between the perfect match (PM) and the mismatch (MM) as detailed in the Affymetrix Genechip procedure (www.Affymetrix.com). A p-value risk was associated with the signal intensity. For each gene, the mean signal expression and standard deviation (SD) were calculated for each group. The fold change was determined by the ratio between old and young, and between “AD-like” and healthy old in order to study gene expression during physiological and pathological aging, respectively. All primary expression data files (*.cel) and (*.xls) are available at the authors' web site (http://www.mmdn.univ-montp2.fr/) and at GEO/NCBI (provisional accession number GSE21779). All steps were conducted according to the MIAME (Minimum Information About a Microarray Experiment) checklist [43].

### Detection of genes that were differently expressed in the three groups

To identify genes differently expressed in relation with brain aging or pathology, filtered data were analyzed with the SAM (Significance Analysis of Microarrays) method [44], which uses permutations of repeated measurements to protect against non-normal data distribution. For that purpose, each group was compared to the other groups using 1,000 permutations. Because each animal may show intrinsic individual variability, the threshold for determining the rate of change was set at 1.5. SAM calculates the false discovery rate (FDR) with a q-value according to the delta cutoffs. The statistical significance of the genes was also investigated by Student *t*-test or by ANOVA with the “R” software. Finally, to construct a predictive model of the changes observed, a projection by principal component analysis (PCA) was carried out.

### Gene profiling

The gene expression profile of each group was established using the Cluster and Treeview software programs [45]. These analyses allowed us to build a hierarchical clustering of the genes that are significantly associated with brain aging.

### Functional categorization of the identified genes

To categorize the genes that were differentially expressed, we used two approaches: the first approach consisted in searching the literature using PubMed (NCBI), GenBank (www.ncbi.nlm.nih.gov/Genbank/), or specific databases such as Gene Expression Omnibus (GEO) (www.ncbi.nlm.nih.gov/geo/), the KEEG pathway database (www.genome.jp/kegg/pathway.html), UniProt (www.uniprot.org), and the Allen Brain Atlas (www.brain-map.org; http://humancortex.alleninstitute.org/has/human/docs.html). The second approach consisted in assigning genes to biological and molecular functions using the hierarchical database of the Gene Ontology (GO) consortium and EASE (Expression Analysis Systematic Explorer software; www.geneontology.org/). Finally, PathwayArchitect (Stratagene, Genome Exploration, UK) and IPA-Ingenuity (www.ingenuity.com) allowed us to build interactive networks.

## Supporting Information

Table S1 Genes sorted by anova
(0.05 MB XLS)

Table S2 Gene functional categorization
(0.19 MB XLS)
